# Pretreatment Systemic Immune-Inflammation Index Can Predict Response to Neoadjuvant Chemotherapy in Cervical Cancer at Stages IB2-IIB

**DOI:** 10.3389/pore.2022.1610294

**Published:** 2022-04-27

**Authors:** Pingping Liu, Yinan Jiang, Xiaojing Zheng, Baoyue Pan, Huiling Xiang, Min Zheng

**Affiliations:** Department of Gynecology, State Key Laboratory of Oncology in South China, Collaborative Innovation Center for Cancer Medicine, Sun Yat-Sen University Cancer Center, Guangzhou, China

**Keywords:** cervical cancer, prognosis, neoadjuvant chemotherapy, pathological complete response, systemic immune-inflammation index

## Abstract

**Background:** The systemic immune-inflammation index (SII) has been identified as a predictor of chemotherapy efficacy for a variety of cancers, and we aimed to determine its ability to predict the response to chemotherapy and its long-term prognosis for patients with cervical squamous cell carcinoma (CSCC) who have underwent platinum-based neoadjuvant chemotherapy (NACT).

**Methods:** The date from 210 patients (133 in the training cohort and 77 in the validation cohort) with CSCC who received NACT were analyzed retrospectively. The association between SII and the pathological complete response (pCR) was determined using Pearson’s chi-square test, receiver operating characteristic (ROC) curve, and Logistic regression analysis. The Kaplan-Meier method and Cox proportional regression model were used to assess the relationship between SII and progression-free survival (PFS) or overall survival (OS).

**Results:** The calculated optimal SII cutoff values for pCR and survival were 568.7051 and 600.5683, respectively, and patients were divided into two groups: a low SII group (≤568.7051 or ≤600.5683) and a high SII group (>568.7051 or >600.5683). A high SII was associated significantly with a lower pCR. Further analysis determined that SII was a more efficient predictor of pCR than the prognostic nutritional index, platelet-to-lymphocyte ratio, and lymphocyte-to-monocyte ratio. Upon multivariate logistic analysis, SII proved to be an independent risk factor to predict the pCR of patients with CSCC. Kaplan-Meier analysis demonstrated that PFS and OS rates were significantly higher in the low-SII group compared with those in the high-SII group. Additional multivariate analysis indicated that the SII is an independent prognostic factor for patients with CSCC treated with NACT.

**Conclusion:** The results confirmed that the pre-treatment SII is not only an independent predictor of pCR but also an independent prognostic factor of CSCC patients treated with platinum based NACT.

## Introduction

Cervical cancer is the most common malignancy of the female reproductive system and has the highest morbidity and mortality rate among all gynecological tumors in China [1, 2]. In recent years, the occurrence of cervical cancer has increased, while the median age of patients has decreased, highlighting cervical cancer as a serious threat to women’s health. Early diagnosis and appropriate treatment are the best ways to optimize patient outcome. For patients with early stage (IB-IIB) cervical cancer, radical surgery is commonly the first choice. However, in cases, namely those with patients with large tumors, where radical surgery cannot achieve satisfactory results, neoadjuvant chemotherapy (NACT) might serve as an alternate and more effective treatment method.

NACT is commonly defined as chemotherapy that is administered to a patient before their operation or radiotherapy with the aim of shrinking the tumor, thus improving its resection rate, reducing the scope of the operation and the injury caused by surgery, and suppressing or eliminating possible micrometastatic foci. NACT can improve patient prognosis, so it is especially preferable for young patients who wish to maintain their fertility. Furthermore, the use of NACT can help to understand a patient’s sensitivity to chemotherapy and provide guidance for postoperative treatment [3]. However, if NACT treatment fails, patients might lose their optimal surgical opportunity. Therefore, it is imperative to find effective and feasible indicators to predict the efficacy of chemotherapy and to ultimately guide the individualized treatment of patients.

Recent research has demonstrated that a patient’s pre-treatment inflammatory and immunological status plays a crucial role in the development of solid malignant tumors [4–6], the relevant indicators of which include the platelet-to-lymphocyte ratio (PLR), the lymphocyte-to-monocyte ratio (LMR), the prognostic nutritional index (PNI), and the systemic immune-inflammation index (SII). Among them, the SII, an integrated indicator based on peripheral lymphocyte, neutrophil, and platelet counts, has been considered to best reflect the balance of host inflammatory and immune status. The SII can also be used to predict the treatment response and survival for a variety of malignant cancers, including breast cancer, rectal cancer, gastric cancer, and pancreatic adenocarcinoma [7–11]. However, the potential utility of the SII on predicted response to NACT for patients with cervical cancer has not been determined.

In this study, the SII in patients with cervical cancer who underwent NACT was evaluated as an indicator for treatment response, and the association between the SII and patient survival was also explored. We found that the SII was a promising, independent, predictive factor for the pathological complete response (pCR) of cervical cancer treated with NACT and might be an independent factor for survival.

## Materials and Methods

### Patient Selection

A total of 210 patients with cervical squamous cell carcinoma (CSCC) who received NACT in our hospital from November 2005 to November 2014 and met the following criteria were included in this study: 1) the patient had been histologically confirmed with CSCC; 2) their Eastern Cooperative Oncology Group (ECOG) performance status was ≤2; 3) their FIGO stage (2009) was IB2-IIB; 4) the patient received preoperative NACT and did not receive antitumor therapy before chemotherapy; 5) blood biochemical examination data was available 7 days before NACT; 6) clinicopathological data was complete; 7) the patient had no history of malignant disease; 8) no factors were present that could affect the results of blood tests, including acute or chronic infection, blood disease, or special drugs taken before treatment.

Our analysis involved two independent patient cohorts: the training cohort, which consisted of 133 patients diagnosed from November 2005 to November 2012, and the validation cohort, which consisted of 77 patients diagnosed from January 2013 to November 2014.

The present study was performed according to the ethical standards of the World Medical Association Declaration of Helsinki and was approved by the Institutional Review Board and Independent Ethics Committee of the Sun Yat-sen University Cancer Center (B2021-417-01).

### Data Collection and Analysis

Demographic, laboratory value, tumor stage, tumor size, and postoperative pathological features including tumor differentiation, lymph node metastasis, and pCR, were retrieved retrospectively from patients’ electronic medical records. Pretreatment laboratory values within 7 days before neoadjuvant therapy were analyzed. The clinical outcomes evaluated included pCR, overall survival (OS) and progression-free survival (PFS) rates. pCR was defined as the absence of viable tumor cells in the evaluated pathological specimen. OS was defined as the interval from the date of chemotherapy until death from any cause or the last follow-up, and PFS was defined as the interval from surgery to disease recurrence, death, or the last follow-up.

From the blood sample laboratory values, the absolute platelet (P), neutrophil (N), and lymphocyte (L) counts were used to calculate the SII (SII = P*[N/L]). The PNI was based on albumin and absolute lymphocyte count; briefly, PNI = serum albumin level (g/L) + 5 × total lymphocyte count (10^9^/L). PLR was defined as the total platelet count divided by the absolute lymphocyte count. LMR was calculated using the following formula: LMR = total lymphocyte count (109/L)/absolute monocyte count (109/L). Optimal cutoff values for SII, PNI, PLR, LMR, platelet, neutrophil, and lymphocyte were calculated individually using a receiver operating characteristic (ROC) curve.

### Surveillance and Statistical Analysis

The follow-up schedule for patients was as follows: Evaluation at 3-month intervals during the first 2 years, 6-month intervals over the next 3 years, and then annually. All patient outcomes were evaluated in March 2021, with the primary endpoint being pCR and secondary endpoints being OS and PFS.

Data were analyzed using IBM SPSS version 22.0 (IBM Corp., Armonk, NY, United States). The ROC analysis was performed to determine the best cutoff values for predicting pCR, and the area under curve (AUC) was used to assess these predictive values (including SII, PNI, PLR, LMR, platelet, neutrophil, and lymphocyte). Logistic regression analysis was performed to determine which independent factors were predictive of pCR. Pearson’s chi-square test was used to assess the association between the SII and other clinicopathological factors. For survival analysis (both OS and PFS), the optimal cutoff values for relative indexes were also calculated by ROC curves according to the OS of patients. All survival curves were plotted using the Kaplan-Meier method and compared using the log-rank test. Cox proportional hazards regression analysis was performed to identify independent factors. *p* ≤ 0.05 indicated statistical significance, and all *p*-values were based on two-sided testing.

## Results

### Patient Characteristics

Data from a total of 210 patients with CSCC, who fit enrollment criteria, were collected for analysis. Among them, 133 patients were placed in the training cohort, while 77 were placed in the validation cohort. The basic characteristics of the patients from these two independent cohorts were listed in Tables 1, 2. All patients underwent platinum-based NACT followed by radical surgery, with a median of two courses of NACT (range, 2–3 courses). In the training cohort, only 18 patients (13.5%) achieved pCR. The median age of the training cohort was 51 years (range, 29–69 years). According to FIGO staging criteria (2009 revision), 64 patients (48.1%) classified as stage IB2, 46 patients (34.6%) as stage IIA2, and 23 patients (17.3%) as stage IIB. The number of cases with high, medium and low differentiation were 4 (3%), 50 (37.6%) and 79 (59.4%), respectively. Additionally, 100 patient cases classified as exogenous tumors (75.2%) and 33 cases as endogenous tumors (24.8%). As for the validation cohort, there were 13 patients (16.9%) achieved pCR. The median age of the training cohort was 48 years (range, 30–64 years). There were 17 patients (22.1%) classified as stage IB2, 50 patients (64.9%) as stage IIA2, and 10 patients (13.0%) as stage IIB. The number of cases with high, medium and low differentiation were 0 (0%), 34 (44.2%) and 43 (55.8%), respectively. Additionally, 72 patient cases classified as exogenous tumors (93.5%) and 5 cases as endogenous tumors (6.5%). Other clinicopathological parameters of these two cohorts are listed in Tables 1, 2.

**TABLE 1:** Clinical characteristics of 133 patients with CSCC divided by pre-treatment SII in the traning cohort.

Characteristics	Patients, n (%)	SII ≤568.7051 (*n* = 51)	SII >568.7051 (*n* = 82)	P
Age (years)				
≤45	41 (30.8%)	13 (25.5%)	28 (34.1%)	0.293
>45	92 (69.2%)	38 (74.5%)	54 (65.9%)	
FIGO stage				
IB2	64 (48.1%)	21 (41.2%)	43 (52.4%)	0.392
IIA2	46 (34.6%)	21 (41.2%)	25 (30.5%)	
IIB	23 (17.3%)	9 (17.6%)	14 (17.1%)	
Histological grade				
G1	4 (3%)	2 (3.9%)	2 (2.4%)	0.830
G2	50 (37.6%)	18 (35.3%)	32 (39%)	
G3	79 (59.4%)	31 (60.8%)	48 (58.5%)	
Tumor size (cm)				
≤5	98 (73.7%)	41 (80.4%)	57 (69.5%)	0.166
>5	35 (26.3%)	10 (19.6%)	25 (30.5%)	
Tumor growth pattern				
Exogenous	100 (75.2%)	38 (74.5%)	62 (75.6%)	0.886
Endogenous	33 (24.8%)	13 (25.5%)	20 (24.4%)	
Lymph node metastasis				
Negative	110 (82.7%)	46 (90.2%)	64 (78%)	0.072
Positive	23 (17.3%)	5 (9.8%)	18 (22%)	
Response to NACT				
pCR	18 (13.5%)	12 (23.5%)	6 (7.3%)	0.008
Non-pCR	115 (86.5%)	39 (76.5%)	76 (92.7%)	
NACT cycles				
≤2	108 (81.2%)	42 (82.4%)	66 (80.5%)	0.789
>2	25 (18.8%)	9 (17.6%)	16 (19.5%)	
Adjuvant treatment				
Yes	123 (92.5%)	46 (90.2%)	77 (93.9%)	0.506
No	10 (7.5%)	5 (9.8%)	5 (6.1%)	
PNI				
≤58.4	118 (88.7%)	41 (80.4%)		
>58.4	15 (11.3%)	10 (19.6%)		
PLR				
≤129.7001	56 (42.1%)	41 (80.4%)		
>129.7001	77 (57.9%)	10 (19.6%)		
LMR				
≤6.2917	107 (80.5%)	36 (70.6%)		
>6.2917	26 (19.5%)	15 (29.4%)		

CSCC, cervical squamous cell carcinoma; SII, systemic immune-inflammation index; NACT, neoadjuvant chemotherapy; pCR, pathological complete response; PLR, platelet-to-lymphocyte ratio; PNI, prognostic nutritional index; LMR, lymphocyte-to-monocyte ratio.

**TABLE 2:** Clinical characteristics of 77 patients with CSCC divided by pre-treatment SII in the validation cohort.

Characteristics	Patients, n (%)	SII ≤568.7051 (*n* = 43)	SII >568.7051 (*n* = 34)	*p*
Age (years)				
≤45	25 (32.5%)	12 (27.9%)	13 (38.2%)	0.336
>45	52 (67.5%)	31 (72.1%)	21 (61.8%)	
FIGO stage				
IB2	17 (22.1%)	12 (27.9%)	5 (14.7%)	0.274
IIA2	50 (64.9%)	27 (62.8%)	23 (67.6%)	
IIB	10 (13%)	4 (9.3%)	6 (17.6%)	
Histological grade				
G1	0 (0%)	0 (0%)	0 (0%)	0.167
G2	34 (44.2%)	16 (37.2%)	18 (52.9%)	
G3	43 (55.8%)	27 (62.8%)	16 (47.1)	
Tumor size (cm)				
≤5	55 (71.4%)	32 (74.4%)	23 (67.6%)	0.514
>5	22 (28.6%)	11 (25.6%)	11 (32.4%)	
Tumor growth pattern				
Exogenous	72 (93.5%)	39 (90.7%)	33 (97.1%)	0.376
Endogenous	5 (6.5%)	4 (9.3%)	1 (2.9%)	
Lymph node metastasis				
Negative	76 (98.7%)	43 (100%)	33 (97.1%)	0.442
Positive	1 (1.3%)	0 (0%)	1 (2.9%)	
Response to NACT				
pCR	13 (16.9%)	12 (27.9%)	1 (2.9%)	0.005
Non-pCR	64 (83.1%)	31 (72.1%)	33 (97.1)	
NACT cycles				
≤2	48 (62.3%)	27 (62.8%)	21 (61.8%)	0.926
>2	29 (37.7%)	16 (37.2%)	13 (38.2%)	
Adjuvant treatment				
Yes	10 (13%)	6 (14%)	4 (11.8%)	0.777
No	67 (87%)	37 (86%)	30 (88.2%)	
PNI				
≤58.4	75 (97.4%)	42 (97.7%)	33 (97.1%)	
>58.4	2 (2.6%)	1 (2.3%)	1 (2.9%)	
PLR				
≤129.7001	26 (33.8%)	21 (48.8%)	5 (14.7%)	
>129.7001	51 (66.2%)	22 (51.2%)	29 (85.3%)	
LMR				
≤6.2917	63 (81.8%)	32 (74.4%)	31 (91.2%)	
>6.2917	14 (18.2%)	11 (25.6%)	3 (8.8%)	

CSCC, cervical squamous cell carcinoma; SII, systemic immune-inflammation index; NACT, neoadjuvant chemotherapy; pCR, pathological complete response; PLR, platelet to lymphocyte ratio; PNI, prognostic nutritional index; LMR, lymphocyte-to-monocyte ratio.

As determined by the ROC curve, the optimal cutoff value of SII for pCR was 568.7051, with an AUC of 0.638, a sensitivity of 66%, and a specificity of 66.7% (Supplementary Figure S1). In the training cohort, the number of cases belonging to the SII≤568.7052 group and SII>568.7051 group were 51 and 82, respectively. As for validation cohort, the cases number of the low SII group and the high SII group were 43 and 34, respectively. The optimal cutoff values for other indexes were as follows: PNI (PNI ≤58.4, PNI >58.4), PLR (PLR ≤129.7001, PNI >129.7001), LMR (LMR ≤6.2917, LMR >6.2917), platelet (platelet ≤226.5, platelet >226.5), neutrophil (neutrophil ≤6.15, neutrophil >6.15), and lymphocyte (lymphocyte ≤2.65, lymphocyte >2.65).

As for survival analysis, we also use the ROC curve to determine the optimal cutoff values for relative indexes. The optimal cutoff value of SII was 600.5683, with an AUC of 0.699, a sensitivity of 88.2%, and a specificity of 47.4% (Supplementary Figure S2). The optimal cutoff values for other indexes were as follows: PNI (PNI ≤49.5, PNI >49.5), PLR (PLR ≤153.4314, PNI >153.4314), LMR (LMR ≤12.25, LMR >12.25), platelet (platelet ≤216, platelet >216), neutrophil (neutrophil ≤5.35, neutrophil >5.35), and lymphocyte (lymphocyte ≤0.87, lymphocyte >0.87).

### Relationship Between Pre-treatment SII and pCR in CSCC

**FIGURE 1:**
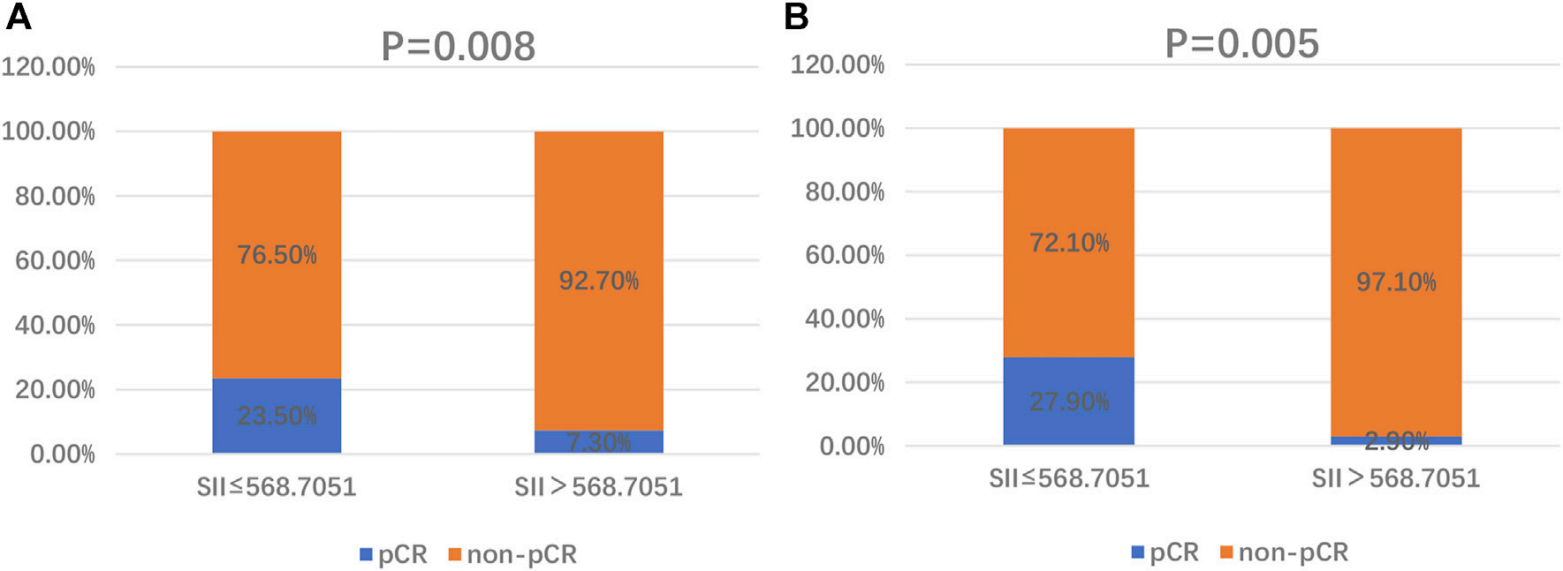
Distribution of pCR and non-pCR in high-SII and low-SII expression groups in the training cohort **(A)** and the validation cohort **(B)**. Abbreviations: SII, systemic immune-inflammation index; pCR, pathological complete response.

**FIGURE 2:**
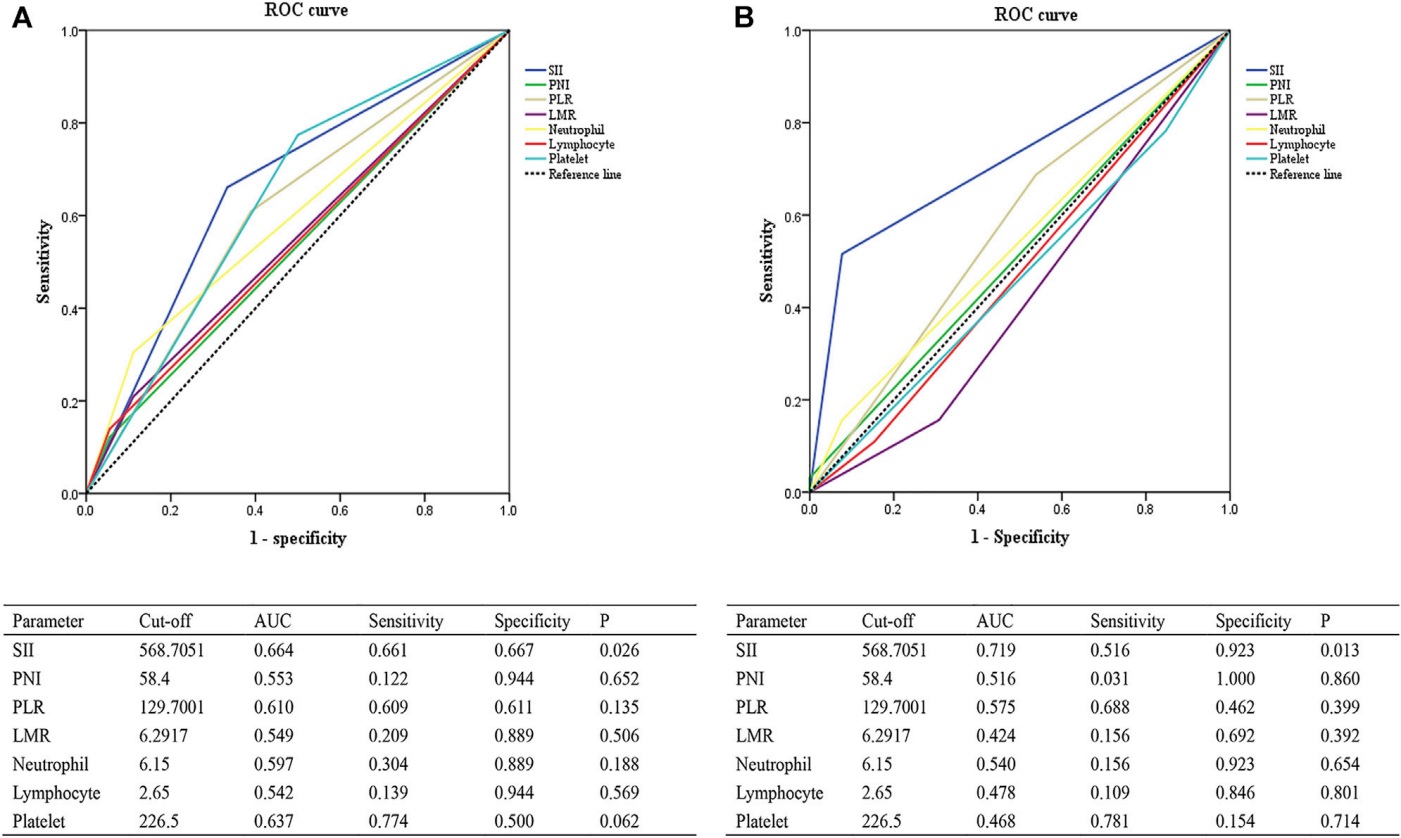
ROC curves evaluating the accuracy of different inflammatory markers for pCR prediction in the training cohort **(A)** and the validation cohort **(B)**. Abbreviations: SII, systemic immune-inflammation index; PNI, prognostic nutritional index; PLR, platelet-to-lymphocyte ratio; LMR, lymphocyte-to-monocyte ratio; AUC, area under curve; ROC, receiver operating characteristic; pCR, pathological complete response.

The associations between the SII and patients’ clinicopathological characteristics are presented in Tables 1, 2. In the training cohort, a negative association was found between SII and pCR: a high SII was significantly associated with a lower pCR rate (SII ≤568.7051 vs. SII >568.7051, 23.5% vs. 7.3%, *p* = 0.008; Figure 1A; Table 1). In the validation cohort, a SII of ≤568.7051 was associated with high pCR rate (SII ≤568.7051 vs. SII >568.7051, 27.9% vs. 2.9%, *p* = 0.005; Figure 1B; Table 2). Further analysis determined that the SII was a more reliable predictor of pCR than PNI, PLR, LMR, platelet, neutrophil, and lymphocyte. When the relationship between various inflammatory indicators and pCR was plotted using a ROC curve, the SII occupied the largest AUC compared with that of PNI, PLR, LMR, platelet, neutrophil, and lymphocyte (*p* = 0.026; Figure 2A and *p* = 0.013; Figure 2B).

### Univariate and Multivariate Analysis of pCR

For the univariate logistic analysis, we included the patients’ age, FIGO stage, histological grade, tumor size, tumor growth pattern, NACT cycles, SII, PNI, PLR, LMR, platelet, neutrophil, and lymphocyte. The results showed that the SII was associated significantly with pCR (odds ratio (OR): 3.897, 95% confidence interval (CI): 1.359–11.174, *p* = 0.011, Table 3 and OR: 12.774, 95% CI: 1.567–104.115, *p* = 0.017, Table 4). We then performed further multivariate logistic analyses of the patients’ FIGO stage, histological grade, tumor size, NACT cycles, SII, and platelet. The results demonstrated that the SII was an independent risk factor to predict the pCR rate for patients with CSCC who were treated with NACT (OR: 3.897, 95% CI: 1.359–11.174, *p* = 0.011, Table 3 and OR: 30.903, 95% CI: 2.152–443.833, *p* = 0.012, Table 4).

**TABLE 3:** Evaluation of the relationship between patient characteristics and pCR using univariate and multivariate analysis in the training cohort.

Variable	Univariate		Multivariate	
	*p*	OR (95% CI)	*p*	OR (95% CI)
Age (≤45 vs. >45 years)	0.399	0.602 (0.185–1.956)		
FIGO stage (IB2-IIA2 vs. IIB)	0.461	1.787 (0.382–8.3721)	0.367	2.086 (0.422–10.324)
Histological grade (G1-G2 vs. G3)	0.501	0.698 (0.245–1.990)	0.340	0.579 (0.189–1.777)
Tumor size (≤5 vs. >5 cm)	0.672	1.292 (0.395–4.225)	0.947	1.044 (0.290–3.754)
Tumor growth pattern (exogenous vs. endogenous)	0.165	2.952 (0.641–13.588)		
NACT cycles (≤2 vs. >2)	0.377	2.000 (0.429–9.323)	0.384	2.044 (0.408–10.233)
SII (≤568.7051 vs. >568.7051)	0.011	3.897 (1.359–11.174)	0.011	3.897 (1.359–11.174)
PNI (≤58.4 vs. >58.4)	0.422	2.356 (0.291–19.105)		
PLR (≤129.7001 vs. >129.7001)	0.086	2.444 (0.882–6.772)		
LMR (≤6.2917 vs. >6.2917)	0.341	2.110 (0.454–9.815)		
Neutrophil (≤6.15 vs. >6.15)	0.107	3.500 (0.763–16.046)		
Lymphocyte (≤2.65 vs. >2.65)	0.342	2.747 (0.342–22.098)		
Platelet (≤226.5 vs. >226.5)	0.018	3.423 (1.232–9.512)	0.219	2.069 (0.649–6.599)

pCR, pathological complete response; NACT, neoadjuvant chemotherapy; SII, systemic immune-inflammation index; PNI, prognostic nutritional index; PLR, platelet-to-lymphocyte ratio; LMR, lymphocyte-to-monocyte ratio; OR, odds ratio; CI, confidence interval.

**TABLE 4:** Evaluation of the relationship between patient characteristics and pCR using univariate and multivariate analysis in the validation cohort.

Variable	Univariate		Multivariate	
	*p*	OR (95% CI)	*p*	OR (95% CI)
Age (≤45 vs. > 45 years)	0.886	0.910 (0.251–3.300)		
FIGO stage (IB2-IIA2 vs. IIB)	0.048	0.233 (0.055–0.989)	0.020	0.059 (0.005–0.641)
Histological grade (G1-G2 vs. G3)	0.651	0.754 (0.222–2.557)	0.795	1.211 (0.285–5.143)
Tumor size (≤5 vs. > 5 cm)	0.261	2.500 (0.506–12.341)	0.545	1.704 (0.303–9.574)
Tumor growth pattern (exogenous vs. endogenous)	0.178	0.270 (0.040–1.810)		
NACT cycles (≤2 vs. >2)	0.575	1.442 (0.401–5.189)	0.339	2.137 (0.451–10.122)
SII (≤568.7051 vs. >568.7051)	0.017	12.774 (1.567–104.115)	0.012	30.903 (2.152–443.833)
PNI (≤58.4 vs. >58.4)	0.999	>1 (0- >1)		
PLR (≤129.7001 vs. >129.7001)	0.305	1.886 (0.561–6.335)		
LMR (≤6.2917 vs. >6.2917)	0.206	0.417 (0.107–1.619)		
Neutrophil (≤6.15 vs. >6.15)	0.466	2.222 (0.259–19.052)		
Lymphocyte (≤2.65 vs. >2.65)	0.651	0.675 (0.124–3.693)		
Platelet (≤226.5 vs. >226.5)	0.601	0.649 (0.129–3.278)		

pCR, pathological complete response; NACT, neoadjuvant chemotherapy; SII, systemic immune-inflammation index; PNI, prognostic nutritional index; PLR, platelet-to-lymphocyte ratio; LMR, lymphocyte-to-monocyte ratio; OR, odds ratio; CI, confidence interval.

### Association Between Pre-Treatment SII and Survival Outcome

The last follow-up for all patients was March 2021, and the median follow-up time for the training cohort and validation cohort was 98 months (range 4–156 months) and 84.6 months (range 13–106 months), respectively. During follow-up for the training cohort, 23 (17.3%) patients experienced tumor recurrence or metastasis, and 21 (15.8%) patients ultimately died from tumor progression. In the validation cohort, tumor progression and death occurred in 11 (14.3%) and 9 (11.7%) patients, respectively.

**FIGURE 3:**
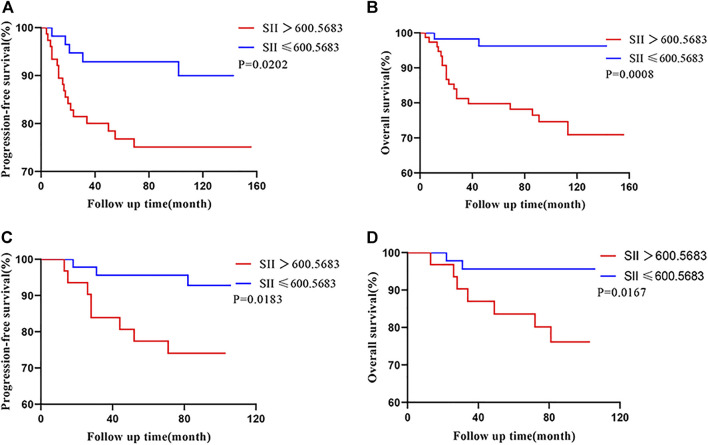
Kaplan-Meier survival curves for 133 patients and 77 patients with CSCC based on SII cut-off values in the training cohort **(A,B)** and the validation cohort **(C,D)**, respectively. Abbreviations: CSCC, cervical squamous cell carcinoma; SII, systemic immune-inflammation index.

Overall, the 5-year PFS and OS rates, in the training cohort, were 84.2% and 87.2%. The patients with a SII ≤600.5683 displayed 5-year PFS and OS rates of 93.0% and 96.5%, respectively, while patients with a SII >600.5683 displayed 5-year PFS and OS rates of 77.6% and 80.3%, respectively. Kaplan-Meier analysis demonstrated that the PFS and OS rates of the low-SII group were significantly higher than those of the high-SII group (*p* = 0.0202 and *p* = 0.0008, respectively; Figures 3A,B).

In the validation cohort, the patients with a SII ≤600.5683 displayed 5-year PFS and OS rates of 95.7% and 95.7%, respectively, while patients with a SII >600.5683 displayed 5-year PFS and OS rates of 80.6% and 83.9%, respectively. Moreover, patients with a low SII had higher PFS and OS relative those with a high SII (Kaplan–Meier analysis, *p* = 0.0183 and *p* = 0.0167, respectively; Figures 3C,D).

### Prognostic Analysis of Clinical Factors

In the training cohort, univariate analysis revealed that SII (HR 3.049, 95% CI: 1.131–8.219, *p* = 0.028), PLR (HR 3.230, 95% CI: 1.396–7.475, *p* = 0.006), FIGO stage (HR 2.808, 95% CI: 1.189–6.631, *p* = 0.018), tumor growth pattern (HR 2.807, 95% CI: 1.238–6.362, *p* = 0.013), and lymph node metastasis (HR 5.601, 95% CI: 2.464–12.734, P < 0.001) were prognostic factors for PFS (Table 5); while a high SII (HR 8.060, 95% CI: 1.876–34.628, *p* = 0.005), high PLR (HR 4.171, 95% CI: 1.679–10.365, *p* = 0.002), and positive lymph node metastasis (HR 9.471, 95% CI: 3.922–22.87, *p* < 0.001) were significant negative predictors of OS (Table 7). In the validation cohort, univariate analysis revealed that SII (HR 4.331, 95% CI: 1.148–16.334, *p* = 0.030) was significantly associated with patient PFS (Table 6), similar findings were observed when OS was used as primary treatment outcome (Table 8).

**TABLE 5:** Univariate and multivariate analysis of prognostic factors for PFS in patients with CSCC in the training cohort.

Variable	Univariate		Multivariate	
	*p*	HR (95% CI)	*p*	HR (95% CI)
Age (≤45 vs. >45 years)	0.555	1.324 (0.522–3.359)		
FIGO stage (IB2-IIA2 vs. IIB)	0.018	2.808 (1.189–6.631)	0.004	3.729 (1.516–9.175)
Histological grade (G1-G2 vs. G3)	0.822	1.101 (0.476–2.545)		
Tumor size (≤5 vs. >5 cm)	0.656	0.798 (0.296–2.150)		
Tumor growth pattern (exogenous vs. endogenous)	0.013	2.807 (1.238–6.362)	0.187	1.752 (0.761–4.031)
Lymph node metastasis (negative vs. positive)	<0.001	5.601 (2.464–12.734)	<0.001	5.092 (2.217–11.694)
NACT cycles (≤2 vs. >2)	0.133	1.978 (0.813–4.811)		
Adjuvant treatment (yes vs. no)	0.227	0.473 (0.141–1.593)		
SII (≤600.5683 vs. >600.5683)	0.028	3.049 (1.131–8.219)	0.039	2.962 (1.057–8.301)
PNI (≤49.5 vs. >49.5)	0.914	0.943 (0.321–2.772)		
PLR (≤153.4314 vs. >153.4314)	0.006	3.230 (1.396–7.475)	0.279	1.761 (0.633–4.901)
LMR (≤12.25 vs. >12.25)	0.146	4.445 (0.596–33.160)		
Neutrophil (≤5.35 vs. >5.35)	0.289	1.557 (0.687–3.530)		
Lymphocyte (≤0.87 vs. >0.87)	0.674	20.622 (0- >1)		
Platelet (≤216 vs. >216)	0.320	1.851 (0.550–6.229)		

CSCC, cervical squamous cell carcinoma; PFS, progression free survival; HR, hazard ratio; CI, confidence interval; NACT, neoadjuvant chemotherapy; SII, systemic immune-inflammation index; PNI, prognostic nutritional index; PLR, platelet-to-lymphocyte ratio; LMR, lymphocyte-to-monocyte ratio.

**TABLE 6:** Univariate and multivariate analysis of prognostic factors for PFS in patients with CSCC in the validation cohort.

Variable	Univariate		Multivariate	
	*p*	HR (95% CI)	*p*	HR (95% CI)
Age (≤45 vs. >45 years)	0.090	0.358 (0.109–1.175)		
FIGO stage (IB2-IIA2 vs. IIB)	0.203	2.370 (0.629–8.937)	0.201	2.623 (0.598–11.495)
Histological grade (G1-G2 vs. G3)	0.562	0.704 (0.215–2.306)	0.847	0.884 (0.251–3.790)
Tumor size (≤5 vs. >5 cm)	0.197	2.184 (0.666–7.160)	0.227	2.120 (0.627–7.164)
Tumor growth pattern (exogenous vs. endogenous)	0.616	1.693 (0.216–13.254)		
NACT cycles (≤2 vs. >2)	0.560	1.424 (0.434–4.667)	0.985	1.013 (0.271–3.790)
Adjuvant treatment (yes vs. no)	0.643	0.615 (0.079–4.803)		
SII (≤600.5683 vs. >600.5683)	0.030	4.331 (1.148–16.334)	0.038	4.090 (1.077–15.526)
PNI (≤49.5 vs. >49.5)	0.673	1.331 (0.353–5.020)		
PLR (≤153.4314 vs. >153.4314)	0.674	1.291 (0.394–4.231)		
LMR (≤12.25 vs. >12.25)	0.787	0.049 (0- >1)		
Neutrophil (≤5.35 vs. >5.35)	0.154	2.443 (0.715–8.351)		
Lymphocyte (≤0.87 vs. >0.87)	—	—		
Platelet (≤216 vs. >216)	0.649	1.612 (0.206–12.604)		

CSCC, cervical squamous cell carcinoma; PFS, progression free survival; HR, hazard ratio; CI, confidence interval; NACT, neoadjuvant chemotherapy; SII, systemic immune-inflammation index; PNI, prognostic nutritional index; PLR, platelet-to-lymphocyte ratio; LMR, lymphocyte-to-monocyte ratio.

In the training cohort, multivariate analysis further indicated that the SII (HR 2.962, 95% CI: 1.057–8.301, *p* = 0.039), FIGO stage (HR 3.729, 95% CI: 1516–9.175, *p* = 0.004), and lymph node metastasis (HR 5.092, 95% CI: 2.217–11.694, *p* < 0.001) were independent predictors of PFS (Table 5); as for OS, the SII (HR 5.171, 95% CI: 1.176–22.733, *p* = 0.030) and lymph node metastasis (HR 6.961, 95% CI: 2.843–17.043, *p* < 0.001) were its independent prognostic factors (Table 7). In the validation cohort, we further performed multivariate analyses of the patients’ FIGO stage, histological grade, tumor size, NACT cycles, and SII, the results indicated that the SII was an independent prognostic predictor for PFS (Table 6). A similar trend was also identified for OS in the validation cohort (Table 8).

**TABLE 7:** Univariate and multivariate analysis of prognostic factors for OS in patients with CSCC in the training cohort.

Variable	Univariate		Multivariate	
	*p*	HR (95% CI)	*p*	HR (95% CI)
Age (≤45 vs. >45 years)	0.206	2.020 (0.680–6.006)		
FIGO stage (IB2-IIA2 vs. IIB)	0.119	2.123 (0.823–5.478)		
Histological grade (G1-G2 vs. G3)	0.111	2.262 (0.828–6.176)		
Tumor size (≤5 vs. >5 cm)	0.411	1.464 (0.590–3.628)		
Tumor growth pattern (exogenous *vs*. endogenous)	0.181	1.825 (0.756–4.406)		
Lymph node metastasis (negative vs. positive)	<0.001	9.471 (3.922–22.87)	<0.001	6.961 (2.843–17.043)
NACT cycles (≤2 vs.>2)	0.232	1.784 (0.691–4.608)		
Adjuvant treatment (yes vs. no)	0.6	0.677 (0.158–2.909)		
SII (≤600.5683 vs. >600.5683)	0.005	8.060 (1.876–34.628)	0.030	5.171 (1.176–22.733)
PNI (≤49.5 vs. >49.5)	0.800	1.171 (0.344–3.980)		
PLR (≤153.4314 vs. >153.4314)	0.002	4.171 (1.679–10.365)	0.650	1.275 (0.447–3.632)
LMR (≤12.25 vs. >12.25)	0.114	5.077 (0.677–38.063)		
Neutrophil (≤5.35 vs. >5.35)	0.064	2.263 (0.953–5.374)		
Lymphocyte (≤0.87 vs. >0.87)	0.681	20.652 (0- >1)		
Platelet (≤216 vs. >216)	0.091	5.655 (0.759–42.151)		

CSCC, cervical squamous cell carcinoma; OS, overall survival; HR, hazard ratio; CI, confidence interval; NACT, neoadjuvant chemotherapy; SII, systemic immune-inflammation index; PNI, prognostic nutritional index; PLR, platelet-to-lymphocyte ratio; LMR, lymphocyte-to-monocyte ratio.

**TABLE 8:** Univariate and multivariate analysis of prognostic factors for OS in patients with CSCC in the validation cohort.

Variable	Univariate		Multivariate	
	*p*	HR (95% CI)	*p*	HR (95% CI)
Age (≤45 vs. >45 years)	0.120	0.352 (0.014–1.312)		
FIGO stage (IB2-IIA2 vs. IIB)	0.483	1.755 (0.356–8.454)	0.528	1.744 (0.311–9.788)
Histological grade (G1-G2 vs. G3)	0.555	0.673 (0.181–2.507)	0.751	0.799 (0.200–3.195)
Tumor size (≤5 vs. >5 cm)	0.282	2.059 (0.552–7.680)	0.372	1.844 (0.480–7.081)
Tumor growth pattern (exogenous *vs*. endogenous)	0.475	2.137 (0.267–17.128)		
NACT cycles (≤2 vs. >2)	0.628	1.384 (0.372–5.158)	0.897	1.100 (0.260–4.654)
Adjuvant treatment (yes vs. no)	0.803	0.768 (0.096–6.140)		
SII (≤600.5683 vs. >600.5683)	0.032	5.560 (1.155–26.776)	0.042	5.143 (1.061–24.927)
PNI (≤49.5 vs. >49.5)	0.500	1.717 (0.356–8.267)		
PLR (≤153.4314 vs. >153.4314)	0.651	1.354 (0.363–5.045)		
LMR (≤12.25 vs. >12.25)	0.729	1.320 (0.274–6.355)		
Neutrophil (≤5.35 vs. >5.35)	0.076	3.295 (0.884–12.277)		
Lymphocyte (≤0.87 vs. >0.87)	—	—		
Platelet (≤216 vs. >216)	0.824	1.266 (0.158–10.125)		

CSCC, cervical squamous cell carcinoma; OS, overall survival; HR, hazard ratio; CI, confidence interval; NACT, neoadjuvant chemotherapy; SII, systemic immune-inflammation index; PNI, prognostic nutritional index; PLR, platelet-to-lymphocyte ratio; LMR, lymphocyte-to-monocyte ratio.

## Discussion

Recent studies have described pre-treatment SII as having a role in predicting the prognosis of solid malignant tumors. However, to the best of our knowledge, no studies have investigated the predictive ability of pre-treatment SII with regard to response to treatment of cervical cancer. In the present study, we demonstrated the significant predictive ability of pre-treatment SII in patients with cervical cancer who were treated with NACT. Our results concluded that a high SII (≤568.7051 vs. > 568.7051) was associated significantly with a decreased pCR to NACT. Logistic regression analysis showed that a SII of ≤568.7051 was an independent predictor for pCR. Moreover, a high pre-treatment SII (>600.5683) was associated significantly with reduced OS and PFS in patients with cervical cancer treated with NACT. Multivariate analysis confirmed the SII as an independent prognostic factor.

The SII was first constructed as a novel index in 2014 and is based on host lymphocyte, neutrophil, and platelet counts [7]; its predictive ability with regard to solid cancers can be explained by the function of these three kinds of cells.

Lymphocytes play an important role in tumor defense by inducing apoptosis of tumor cells through immune surveillance, thereby inhibiting cancer cell invasion, proliferation, and metastasis. Denkert et al. [12] reported that an increased concentration of tumor-infiltrating lymphocytes (TIL) could be used to predict the response to NACT in patients with breast cancer across all molecular subtypes. Furthermore, TILs were associated with survival benefit in both HER2-positive and triple-negative breast cancers. D’Alessandris et al. [13] also found that higher TIL infiltration correlated with a higher pCR rate in patients with cervical cancer treated with NACT. Meanwhile, decreased proportions of tumor-infiltrating CD4+T cells were found to be closely related to tumor progression and lymph node metastasis in cervical carcinoma [14]. The cell numbers of lymphocyte subsets, including CD4^+^, CD8^+^, CD3^+^, and CD56 + T cells, are decreased in patients with advanced cancer, leading to weakened lymphocyte-mediated anti-tumor immune responses [15]. Therefore, lymphopenia is deemed to be an independent prognostic factor for patient survival in several cancers.

In contrast to lymphocytes, neutrophils have a significant tumor-promoting effect. In the tumor microenvironment, neutrophils can derive subsets of myeloid-derived suppressor cells (MDSCs), which, in circulation, can produce reactive oxygen species and arginase, suppressor of T lymphocytes, thereby causing a strong immunosuppressive effect. In addition, the lysis of the neutrophils’ nuclear membrane and the release of their nuclear DNA forms neutrophil extracellular traps (Nnets), which transport circulating tumor cells (CTC) to the metastatic site for further growth [16–18]. Neutrophils can also release matrix metalloproteinase-9 (MMP9), vascular endothelial growth factor (VEGF), and inflammatory mediators, such as interleukin 6(IL-6) and tumor necrosis factor-β (TNF-β), which promote the invasion, proliferation and distant metastasis of tumor cells [19]. Gentles et al. [20] analyzed immune cells in 14 types of solid tumors and found that neutrophils were the cell group with the most unfavorable prognosis in patients with tumors. Murakami et al. [21] found that patients with gastric cancer with higher levels of peripheral blood neutrophils had a poor response to chemotherapy, and that their OS was shorter by an average of 8 months compared with patients with lower neutrophil counts. In cervical cancer, a high degree of neutrophil infiltration within the tumor is associated with resistance to radiotherapy and is a factor for poor prognosis [22, 23].

Increased platelets due to thrombosis, which is common in patients with tumors, can promote tumor angiogenesis and assist the immune escape of tumor cells [24]. Meanwhile, platelets can also induce the production of CTCs, and their secretion of transforming growth factor-β (TGF-β) and platelet-derived growth factor (PDGF) can promote the epithelial-mesenchymal transformation of CTCs [25]. Therefore, platelets also play an important role in promoting tumor progression.

As a comprehensive indicator, the SII can reflect host immune and inflammatory status in a more extensive manner than other indicators. A growing body of data point to the SII being a predictor of chemotherapy efficacy for a variety of cancers. Jiang et al. [8] retrospectively analyzed 387 female patients with breast cancer who were treated with NACT followed by surgery and determined that the SII was an independent predictor of pCR for patients with breast cancer, with the low-SII group showing the highest pCR rate. Eraslan et al. [9] studied 188 patients diagnosed with locally advanced rectal cancer who received neoadjuvant chemoradiotherapy (NACRT) and found that among several inflammatory indices, including neutrophil-to-lymphocyte ratio (NLR), PLR, and SII, only the SII, at a value of <748, was an independent predictive factor of pCR after NACRT (OR: 0.471, 95% CI: 0.224–0.991, *p* = 0.047). Murthy et al. [11] found that the SII could reflect treatment response and outcome following NACT for patients with pancreatic adenocarcinoma. However, no relevant research has yet been reported regarding the predictive value of the SII for response to chemotherapy for patients with cervical cancer.

The results of the present study showed that patients with cervical cancer with an SII of ≤568.7051 before NACT exhibited a higher pCR rate. Additionally, we found that the SII was a better predictor of pCR than PNI, PLR, LMR, platelet, neutrophil, and lymphocyte. In the logistic regression analysis, the SII was an independent predictor of pCR for patients with cervical cancer treated with NACT.

Recently, the pre-treatment SII was demonstrated to predict the prognosis of solid malignant tumors. Dong et al. [26] analyzed a total of 12 studies published between 2016 and 2019 and showed that a high SII was associated significantly with poorer OS and PFS (*p* = 0.001) for colorectal cancer. Jomrich et al. [27] studied 320 consecutive patients undergoing esophagectomy and found that in patients with gastroesophageal junction adenocarcinoma, a high SII was associated significantly with lower survival in patients undergoing neoadjuvant treatment. Zeng et al. [28] revealed that the SII is an important independent prognostic index for pulmonary sarcomatoid carcinoma. Aziz et al. [29] assessed 590 patients with resectable pancreatic ductal adenocarcinoma (PDAC) retrospectively and identified an SII >900 as an independent predictor of cancer-specific survival and recurrence. Hu et al. [7] studied 133 patients with hepatocellular carcinoma who underwent resection and concluded that a high SII (cut-off > 330*10^9^/L) was a potent prognostic indicator of poor outcome. The results were further validated in a prospective study involving 123 patients. In the present study, the optimal cutoff value for SII, calculated using the ROC curve, was 600.5683, and we also determined that a low pretreatment SII was associated significantly with increased OS and PFS in patients with cervical cancer treated with NACT. Moreover, the patients’ pre-treatment SII was an independent prognostic factor in multivariable analysis.

Despite this study’s successful demonstration that pre-treatment SII is an independent predictor of patients’ response to NACT, as well as an independent prognostic factor for cervical cancer, it still had some limitations. First, the hematological data for each patient was collected within 1 week before receiving NACT; however, the value of inflammatory indexes might be affected by various pathological conditions and might vary with time. Second, our study was a retrospective, single center study with a limited number of patients (*n* = 133) and thus might contain selection bias; therefore, a multicenter study with a larger sample size is warranted.

## Conclusion

The present study demonstrated the predictive ability of the pre-treatment SII for patients with cervical cancer after NACT. Our results confirmed that the SII qualifies as an independent predictor of pCR and as an independent prognostic factor for cervical cancer. The low pCR rate and short survival time of patients with high SII scores might be caused by poor chemotherapy sensitivity and, as such, should be managed with other treatments, such as concomitant chemotherapy and radiotherapy. In conclusion, SII measurement will aid patients in their clinical treatment selection, which is bolstered by the fact that measurement of pre-treatment SII is available, inexpensive, and reliable for cervical cancer in clinical practice. Further investigations are warranted to validate these results.

## Data Availability

The datasets presented in this article are not readily available due to patient privacy requirements. Requests to access the datasets should be directed to the corresponding author.

## Supplementary Material

The Supplementary Material for this article can be found online at: https://www.por-journal.com/articles/10.3389/pore.2022.1610294/full#supplementary-material

Supplementary Figure S1 ROC curve analyzing the predictive value of SII for the pCR of patients with CSCC. Notes: AUC, area under the ROC curve; * indicates the cutoff value. Abbreviations: ROC, receiver operating characteristic; pCR, pathological complete response; CSCC, cervical squamous cell carcinoma; SII, systemic immune-inflammation index.

Supplementary Figure S2 ROC curve analyzing the predictive value of SII for the OS of patients with CSCC. Notes: AUC, area under the ROC curve; * indicates the cutoff value. Abbreviations: ROC, receiver operating characteristic; OS, overall survival; CSCC, cervical squamous cell carcinoma; SII, systemic immune-inflammation index.

## Abbreviations

AUC, area under curve; CI, confidence interval; CSCC, cervical squamous cell carcinoma; CTC, circulating tumor cells; ECOG, Eastern Cooperative Oncology Group; HR, hazard ratio; IL-6, interleukin 6; LMR, lymphocyte-to-monocyte ratio; MDSCs, myeloid-derived suppressor cells; MMP9, matrix metalloproteinase-9; NACRT, Neoadjuvant Chemoradiotherapy; NACT, neoadjuvant chemotherapy; NLR, neutrophil-to-lymphocyte ratio; Nnets, neutrophil extracellular traps; OR, odds ratio; OS, overall survival; pCR, pathological complete response; PDAC, pancreatic ductal adenocarcinoma; PDGF, platelet-derived growth factor; PFS, progression-free survival; PLR, platelet-to-lymphocyte ratio; PNI, prognostic nutritional index; ROC, receiver operating characteristic; SII, systemic immune-inflammation index; TGF-β, transforming growth factor-β; TILs, tumor-infiltrating lymphocytes; TNF-β, tumor necrosis factor-β; VEGF, vascular endothelial growth factor.
