# Supplementary figures and images for: Pretreatment Systemic Immune-Inflammation Index Can Predict Response to Neoadjuvant Chemotherapy in Cervical Cancer at Stages IB2-IIB
Source: Pathol Oncol Res. 2022 Apr 27;28:1610294. doi: 10.3389/pore.2022.1610294 (PMC9092215; doi:10.3389/pore.2022.1610294)

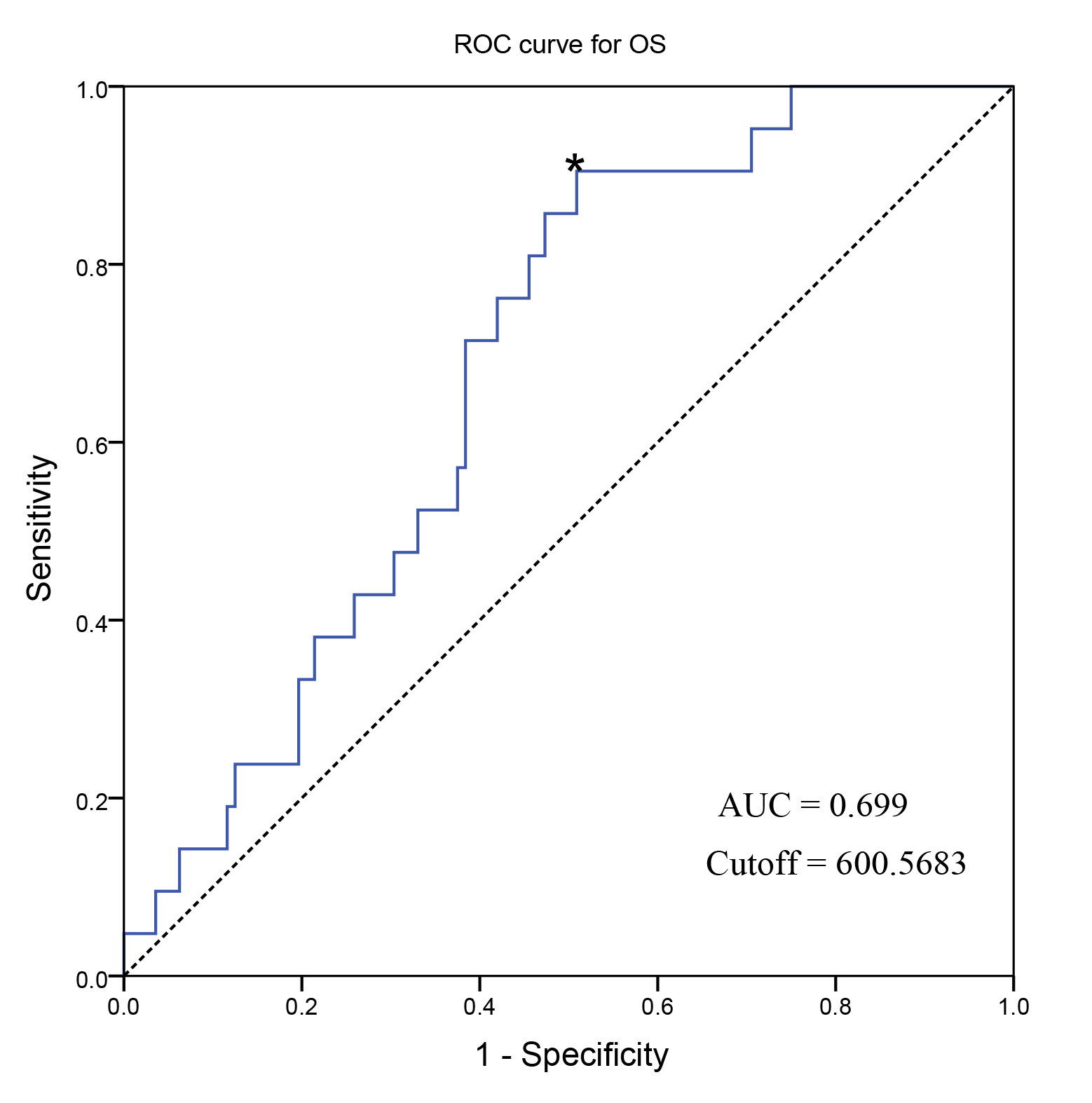

Supplement: Supplementary file 1 [file Image2.TIF]

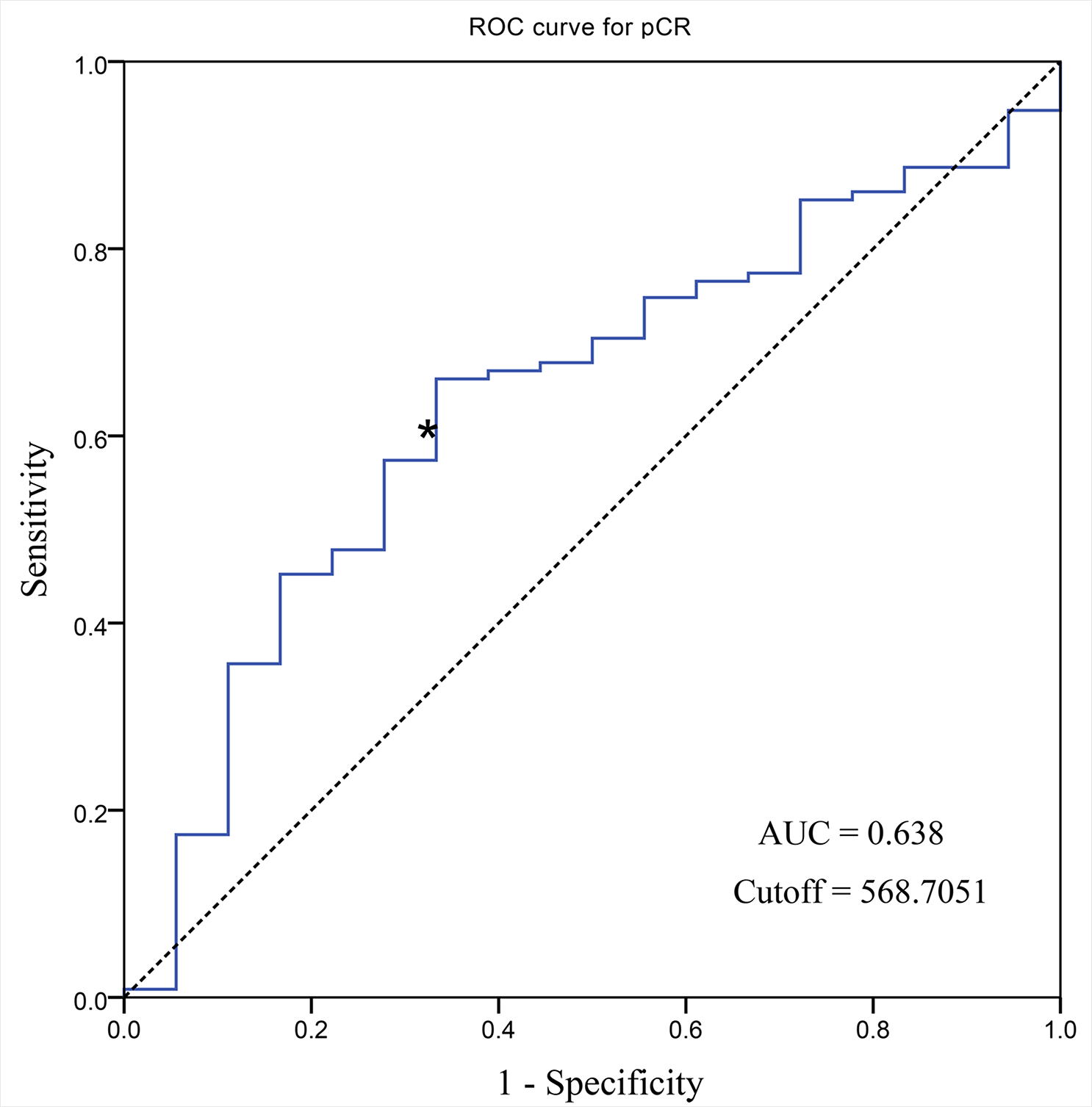

Supplement: Supplementary file 2 [file Image1.TIF]
